# Angiosarcome métastatique de la cuisse: complication rare du lymphoedème

**DOI:** 10.11604/pamj.2015.22.70.8000

**Published:** 2015-09-28

**Authors:** Redouane Ouakrim, Mustapha Mahfoud

**Affiliations:** 1 Service de Chirurgie Orthopédique, CHU Ibn Sina, Université Mohammed V, Rabat, Maroc

**Keywords:** Angiosarcome, lymphoedème, tumeur, métastase, Angiosarcoma, lymphedema, tumor, metastasis

## Image en medicine

Les angiosarcomes sont des tumeurs endothéliales malignes représentant moins de 1% des sarcomes. Dans 10 à 15% des cas ils surviennent sur des zones préalablement irradiées ou sur lymphœdème. Cliniquement, ils se manifestent par placardsou nodules cutanés, infiltrés, érythémateux ou ecchymotiques,parfois associés à des lésions bulleuses séro-hématiques et des ulcérationsdouleureuses. Le diagnostic estconfirmé par l'histologie, qui montre une prolifération de cellules endothéliales atypiques identifiée par immunohistochimie. Le pronostic reste trèsmauvais, quel que soit le traitement. Les lésions sont souvent multifocales d'emblée ou dans les mois qui suivent le diagnostic et lesmétastases sont surtout pulmonaires. Le traitement repose sur la chirurgie et la radiothérapie. La chimiothérapie est réservée aux formes inopérables et métastatiques. Nous rapportons le cas d'une patiente de 76 ans, consultait pour masse tumorale de la face interne de la cuisse gauche sur un terrain de lymphoedème chronique. La biopsie de la masse confirmait le diagnostic d'angiosarcome. Le bilan d'extension révélant des métastases pulmonaires et hépatiques. La patiente est décédée 2 mois après le diagnostic de sa maladie.

**Figure 1:**
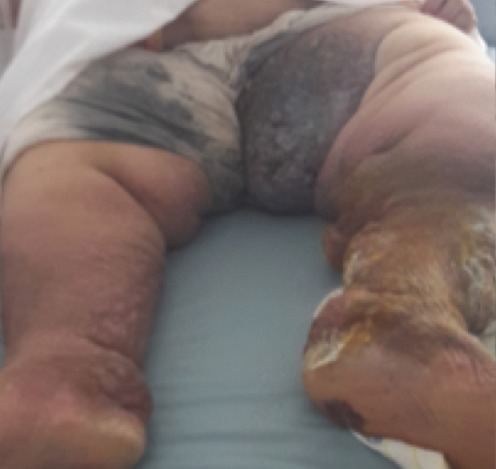
masse tumorale angiomateuse de la cuisse

